# Progressive Supranuclear Palsy—Parkinsonism Predominant (PSP-P)—A Clinical Challenge at the Boundaries of PSP and Parkinson's Disease (PD)

**DOI:** 10.3389/fneur.2020.00180

**Published:** 2020-03-10

**Authors:** Piotr Alster, Natalia Madetko, Dariusz Koziorowski, Andrzej Friedman

**Affiliations:** ^1^Department of Neurology, Medical University of Warsaw, Warsaw, Poland; ^2^Department of Neurology, Wrocław Medical University, Wrocław, Poland

**Keywords:** PSP-P, PSP-RS, progressive supranuclear palsy, neuroimaging, differentiation

## Abstract

Progressive Supranuclear Palsy (PSP) and Parkinson's Disease (PD), especially in their early stages, show overlapping clinical manifestations. The criteria for the diagnosis of PSP, released in 2017, indicate four basic features of the disease—postural instability (P), akinesia (A), oculomotor dysfunction (O) and cognitive and lingual disorders (C), which clarify the interpretation of the disease. There is growing interest in the second most common variant of PSP—parkinsonism predominant PSP-P. It is observed in up to 35% of cases. The diagnosis of PSP-P requires the presence of *akinetic-rigid predominantly axial and levodopa resistant parkinsonism* (A2) or *parkinsonism with tremor and/or asymmetric and/or levodopa responsive* (A3). The development of supplementary methods of examination added new insights to observations related to PSP-P. Among the methods recently analyzed are freezing of swallowing and speech breathing assessment, transcranial sonography, and various methods using magnetic resonance imaging, such as pons/midbrain area ratio and magnetic resonance parkinsonism index (MRPI), fractional anisotropy or mean diffusivity. The proper examination of overlapping parkinsonian syndromes, regardless of the development of the method of examination, remains an incompletely explored issue. The aim of this review is to elucidate which factors may be interpreted as influential in the differential diagnosis of PSP-P, PSP-RS and postural instability and gait difficulty (PIGD) subtype of Parkinson's disease (PD).

## Introduction

Progressive supranuclear palsy (PSP) is the most common atypical parkinsonism. The neuropathology of this tauopathy is related to tufted astrocytes. PSP was first described by, Steele et al. (1). Observations concerning this disease led to the description of various phenotypes. The PSP—Richardson-Steele-Olszewski (PSP-RS)—clinically related with significant oculomotor dysfunction and postural instability in the early stages is the most common (2). The parkinsonism variant of PSP (PSP-P), associated with a less severe course of the disease, is the second most common and is diagnosed in 14–35% cases of PSP (3). The diagnosis of PSP-P is often made retrospectively. Unlike PSP-RS, PSP-P is not associated with the atrophy of midbrain tegmentum, especially in its early stages (4). During the course of the disease, PSP-P may evolve into PSP-RS. Recent criteria for the diagnosis of PSP stress 4 basic factors—oculomotor dysfunction (O), postural instability (P), akinesia (A), and cognitive impairments and language disorders (C), which describe phenotypes of the disease. The intensity of symptoms is graded from 1 to 3 within each factor. Probable PSP-P is associated with *vertical supranuclear gaze palsy* (O1) or *slow velocity of vertical saccades* (O2) and *parkinsonism, akinetic-rigid predominantly axial and levodopa resistant* (A2) or *parkinsonism, with tremor and/or asymmetric and/or levodopa responsive* (A3). Possible PSP-P symptoms may include postural instability and cognitive or language deficits. The presence of A2 and A3 is obligatory (2). The clinical manifestation of PSP subtypes present significant overlaps, however they are associated with differences in neuropathological examination (5–7). The mentioned above features of PSP-P lead to additional difficulties in the differentiation of PSP-P and postural instability gait disorder (PIGD) variant of PD.

## Clinical Examination

A study analyzing phenotypes of PSP using Dementia Rating Scale-2, Frontal Assessment Battery, and lexical fluency scale, revealed more rapid deterioration in PSP-RS. The phenotype of the disease was interpreted as a predictor of the course of the disease. The authors of the study indicated that freezing of gait was more frequent in PSP-P whereas bradyphrenia was more frequent in PSP-RS. Other clinical features, e.g., dysarthria and falls, were similar in both phenotypes (8).

A study, based on examination of 25 patients with PSP in Yonago, showed increased prevalence of PSP in Japan. Twelve percentage of them had a clinical manifestation of PSP-P. PSP-P patients in this study were associated with clinical Parkinson's Disease-like syndrome. The authors of the study interpreted asymmetric onset, tremor and primary levodopa treatment response as elements of the clinical manifestation of PSP-P. Additionally, the mean duration of the disease within patients with PSP-P was longer−7.7 years compared with 3.8–4.0 for patients with either probable or possible PSP-RS (9). The weakness of this study was low number of patients examined and disproportionate groups−3 with PSP-P and 20 with PSP-RS.

In another study, where the mean ages of patients with PSP-P and PSP-RS were similar, the authors observed a significant difference in executive function and processing speed between PSP-RS and brainstem predominant PSP phenotypes—PSP-P and PSP-pure akinesia and gait freezing (PSP-PAGF). Deterioration was more significant in PSP-RS. Other neuropsychological features examined—such as memory, language, working memory, and visuospatial memory—showed no significant differences. This study was based on 3 patients with PSP-P and 14 with PSP-RS (10). Another work presented a 24 month clinical assessment of patients with PD, PSP-RS or PSP-P (11). Out of 180 patients, only 11 were diagnosed with PSP-P and 14 with PSP-RS. The authors showed that verbal fluency deficits and apathy are more characteristic for PSP-RS and PSP-P than PD. Additionally, verbal fluency deficits were found to be more typical of PSP-RS whereas PSP-P was more closely associated with apathy. The research presents a cross sectional neurological, neuropsychiatric, and neuropsychological study. The weakness of the study was the small group of patients with PSP and the disproportion between the PD and PSP groups (11).

In another work evaluating 17 patients with PSP-RS, 12 patients with PSP-P and 30 healthy volunteers, participants were assessed using *Unified Parkinson's Disease Rating Scale-III, PSP rating scale, Berg balance scale, Tinetti performance-oriented mobility assessment and total score, dynamic posturography and MRI*. The study showed that abnormalities regarding balance and those observed in MRI were more significant in PSP-RS. Additionally the qualitative and quantitative assessment of abnormalities, observed in balance deficits, were more related with midbrain atrophy among PSP-P patients (12).

Another work described freezing of swallowing (FOS) among patients with PSP-P. The observation was based on three patients with a diagnosis of probable PSP-P who suffered significant weight loss. Patients underwent videofluoroscopic examination of swallowing (13). The research did not provide any comparison of FOS in PD and PSP-RS. The fluency of speech in the course of dysarthria in PSP was analyzed in a different study, where the pace stability of speech was examined among 16 patients with a clinical diagnosis of PSP-RS, 20 with PSP-P, 60 with PD and 32 healthy volunteers. Each patient was asked to repeat one syllable. Every participant examined in the study repeated the syllable at an individual isochronous pace. The pace stability of speech was assessed by obtaining the coefficient of variance (COV) of interval length in the patients' speech. The comparison of COV indicated more significant intervals in PSP-RS than in PSP-P. Additionally, the results of PSP-P patients were worse than of those with PD. Another feature examined in the study—speech breathing, evaluated by asking the patients to keep saying one of the vowels on one breath, showed worse mean results among patients with PSP-P than with PSP-RS (14).

Another study evaluated speech acoustic analysis as a possible tool for PSP-P differentiation (15). In this work the vocal test was composed of four complex sentences to be read. The authors reported differences between patients with PSP and PD—reduction of NSR (net speech rate—total speech time, without pauses) and increase of PR% (pause ratio—percentage of time consumed by pauses) were observed in PSP. Differences in gender-matching subgroups of PSP and PD patients were observed, however no significant disparities were found between PSP-P and PSP-RS subpopulations. Nevertheless, this study is limited by the number of patients examined (14 with PSP-RS, 12 with PSP-P, 30 with PD) and acoustic parameters.

Faster deterioration of the condition of patients with PSP-RS is correlated with a shorter survival time compared to PSP-P (16, 17). In a study examining 51 patients with PSP-RS, 21 with PSP-P and 49 with multiple system atrophy—parkinsonian type (MSA-P), the mean survival time of patients with PSP-RS was 6.8 years compared to 11.2 years in patients with PSP-P. Additionally the survival time of patients with PSP-P was significantly longer than in MSA-P groups (7.9 years) (16). The uncharacteristic clinical manifestation of PSP-P and various symptomatological overlaps of PSP-P and Dementia with Lewy Bodies, PD, MSA and Vascular Parkinsonism led to a search for possible differentiating factors. The authors of one of the studies indicated visual hallucinations, drug induced dyskinesias and autonomic dysfunction as possibly exclusive features of PSP-P (18).

The most significant clinical and biochemical differences between PSP-P and PSP-RS are associated with the first 2 years of the disease. One of the researches showed that the entities were distinguishable during the early stage (19). PSP-RS patients showed faster deterioration, however, during the course of the disease, features such as postural instability, supranuclear gaze palsy and cognitive impairment became similar. Additionally, tau levels in the cerebrospinal fluid of patients with PSP-RS and PSP-P did not show any significant differences (19).

The clinical examination features of PSP-RS and PSP-P show that in most studies the most significant overlaps between the diseases are observed among patients with longer disease duration. During the first years PSP-P and PD may be undistinguishable. Most of the recently published studies, as the one performed by Borm et al. indicate PIGD as a challenging feature in the differentiation of parkinsonisms. Authors of this study indicate “bedside” PIGD tests as possible methods of differentiation between PD and atypical parkinsonisms. In this study authors bring up the issue of only of the variants of PSP—PSP-RS. The issue considering differentiation of PSP-P and PD is not discussed (20).

The general conclusion of the studies presented in this section of the review is that PSP-P, though associated with a less severe course of the disease, should be interpreted as a more gradually evolving PSP, whereas PSP-RS more dynamically. The vast majority of the studies presented in this section lacked neuropathological examination (9–16, 19). Only the works performed by O'Sullivan and Williams were neuropathologically confirmed (17, 18).

Except for the works conducted by Shoeibi et al., Borm et al., and Lee et al., all of the studies presented in this section of the manuscript were published before the release of current criteria of diagnosis of PSP.

## Additional Examination

A 4-year prospective clinical study, examining 110 patients with an initial diagnosis of PD, evaluated possible imaging biomarkers of vertical gaze palsy. During the 4 year follow up vertical gaze palsy was observed in 10 patients. All patients were assessed using pons/midbrain area ratio 2.0 and magnetic resonance parkinsonism index 2.0 (MRPI 2.0) *(pons area-midbrain area ratio multiplied by middle cerebellar peduncles width-superior cerebellar peduncles* (SCP) *width ratio multiplied by third ventricle width/frontal horns width ratio)* (21, 22). The results of the study revealed significant differences of MRPI 2.0 between PD and PSP-P. Additionally at the end of the 4 year follow up pons/midbrain (P/M) ratio 2.0 as well as MRPI 2.0 distinguished PD and PSP with increased values in PSP-P compared to PD. The MRPI 2.0 was interpreted as a predictor of the phenotype of parkinsonian syndrome (21). Another study presenting 12 patients with PSP-RS, 12 patients with PSP-P and 23 with PD examined midbrain atrophy. Primary MRI was followed by further examination after a minimum of 2 years. Each image was evaluated using pontine to midbrain tegmental areas ratio (P/M ratio) and the length between the *interpeduncular fossa and the center of the cerebral aqueduct at the midmammillary-body level* (MTEGM). Primary examination revealed increased P/M ratio and lower MTEGM in patients with PSP-RS compared to those with PSP-P or PD. During the course of the disease no abnormalities of P/M ratio and MTEGM among patients with PD were detected. Eventual results of patients with PSP-P showed increased P/M ratio and decreased MTEGM. The most significant changes in P/M ratio and MTEGM were found PSP-RS group (23). At this stage no differences between PSP-P and PD were detected.

In a study assessing 10 patients with PSP-RS, 10 with PSP-P, 25 with PD and 24 healthy controls using 3D T1-weighted images, authors evaluated the pons/brain and MRPI. Both pons/brain ratio and MRPI revealed significant differences between PSP-RS and PD as values of the parameters were higher in PSP-RS. Only the pons/brain ratio distinguished between PSP-P and PD as it was increased in PSP-P. The high specificity of this factor−96% was affected by a relatively low sensitivity−60% (24). The lack of significant differences between PSP-P and PD in MRPI shows higher involvement of infratentorial structures in PSP-RS than in PSP-P.

A work presenting 24 patients with PSP-P, indicated that MRPI precedes vertical supranuclear gaze palsy (VSGP) among patients with PSP-P. Patients were assessed every 6 months until the initiation of VSGP (25).

Another study showed differences within the SCP in MRI planimetric assessment. The more significant atrophy of SCP in PSP-RS compared to PSP-P was associated with the fact that postural instability is a more conspicuous element of PSP-RS symptomatology. The study was based on 48 patients with a diagnosis of probable PSP-RS and 30 patients with probable PSP-P. Additionally, the authors of the study evaluated 37 patients with PD. The severity of clinical manifestation in PSP-P (motor and cognitive function, levodopa responsiveness) was interpreted as intermediate between PD and PSP-RS. The deterioration was more pronounced in PSP-RS (26).

3T Magnetic Resonance Imaging (MRI) was analyzed among 23 patients with PD, 12 with PSP-RS and 12 with PSP-P. Authors of one of the studies evaluated mean diffusivity, fractional anisotropy, gray and white matter volume. The authors of the study found significant differences between PSP phenotypes and PD within mesencephalic tegmentum, superior cerebellar peduncle, decussation of SCP and dentate nucleus. Significant abnormalities in PSP were observed in those regions in fractional anisotropy decrease, volume loss and increased mean diffusivity. Additionally, microstructural analysis of the dentatorubrothalamic tract revealed more significant volume loss in PSP-RS than in PSP-P. The decussation of SCP and the thalamus was more marked in PSP-RS than in PSP-P (27). A different study examining 8 patients with the diagnosis of probable PSP-P and 2 with possible PSP-P was conducted using diffusion tensor (DT) MRI. The follow-up duration was 1.6 years and results were compared with 36 age matched healthy controls. Analysis of DT MRI revealed significant damage of supratentorial tracts within the white matter (WM). No deterioration was observed within the cerebellar WM. The authors of the study interpreted the damage of WM supratentorial tracts as a possible marker of PSP-P evolution. The paper did not show any correlation between the clinical manifestation and DT MRI abnormalities (3). In a work highlighting the role of DT MRI 21 patients with PSP-RS, 16 patients with PSP-P and 42 healthy volunteers were examined. The most significant deterioration of infratentorial white matter and thalamic radiations was observed in PSP-RS patients, whereas PSP-P patients were less affected. Authors of the study also indicated the role of combined DT MRI and MRPI assessment, which was interpreted as a possible distinguishing tool of PSP-RS and PSP-P (28).

In a study examining 18 patients with PSP-RS, 14 with PSP-P, 20 with PD and 25 healthy controls, authors verified the role of corpus callosum volumetry in the differentiation of parkinsonian syndromes. The work revealed that the volume of the central part of corpus callosum (CC) was lower in PSP-RS than in PSP-P and PD. The volume of the midanterior part of CC was lower in PSP-RS than in PSP-P and PD, however for PSP-P it was comparable with early PD. The study, though indicating a factor possibly differentiating PSP-RS and PSP-P, did not show any location distinguishing PSP-P from PD (29).

Differences between PSP-RS and PSP-P were also assessed in the context of deficits within the dopaminergic system using dopamine transporter scan (DaTScan) and iodobenzamide scan. In one of the works examining 6 patients with PSP-RS, 4 with PSP-P and 10 with PD decreased striatal uptake in PSP-RS compared to PSP-P was observed in DaTScan and iodobenzamide scan. However, only the results obtained using iodobenzamide scan and not in DaTScan showed statistical significance. This observation was not confirmed in DaTScan. Additionally putamen to putamen-to-caudate nucleus ratio (*putamen counts–occipital cortex counts)/(caudate nucleus counts–occipital cortex counts*) (P/C) revealed significant differences between all subtypes of PSP and PD as it was increased in PSP. No differences between PSP-RS and PSP-P in putamen-to-caudate nucleus ratio were found (30).

Transcranial sonography (TCS) was evaluated among 11 patients with PSP-P and 21 patients with PSP-RS. Hyperechogenic substantia nigra was observed among 14% of patients with PSP-RS and 73% with PSP-P. Hyperechogenic lenticular nucleus on either one or both sides was detected in 67% patients with PSP-RS and PSP-P. Additionally, the authors of the study measured the width of the third ventricle, which tended to be significantly wider within the PSP-RS group (31). Similar observations in the context of the width of the third ventricle were also found in a study, where the research groups consisted of 27 patients with PSP-RS and 7 patients with PSP-P. Hyperechogenic substantia nigra was observed in 86% of patients with PSP-P. On other hand, lack of substantia nigra echogenicity abnormalities was indicated among 96% patients with PSP-RS (32).

Transcranial Magnetic Stimulation (TMS) was analyzed among 15 patients with PSP-RS and 11 with PSP-P. The results of patients with PSP were compared to 15 PD patients (33). Transcallosal inhibition was more visible in PSP-RS than in PSP-P or PD. The work showed that transcallosally projecting output neurons are more affected in PSP-RS than in the related diseases (33). The issue was also brought up in a study by Benussi et al., where authors analyzed the *inhibition of the motor cortex induced by stimulation of the cerebellum with a figure of eight-coli*. The outcome of the work showed significant differences of PSP patients when compared with DLB,CBS, DLB and healthy patients. Each group consisted of 15–26 patients (34). In a work highlighting the use of TMS as method of increasing cerebellar-brain inhibition, authors concluded that it may be used as a treatment of speech and postural disturbances in PSP. Authors did not elaborate on the issue of possible different outcomes of treatment among PSP-P and PSP-RS patients. The work was based on the analysis of 2 patients (35).

A cohort study examining 9,369 patients between 1914 and 2010 revealed 269 patients with a clinical diagnosis of parkinsonian syndrome at the moment of death. The clinical diagnosis was verified by neuropathological examination, which indicated idiopathic PD in 62.2% patients. Neuropathological diagnosis of PSP was confirmed in 4.2%, whereas MSA was detected in 2.3%. The diagnostic specificity of PD rose gradually from 71.2 to 85.7% over the period of research (36). The study did not stress the issue of PD/PSP-P differentiation.

Seventeen pathologically confirmed patients with PSP-RS and 7 with PSP-P were examined using a validated point-counting technique. Cortical volume loss was a parameter distinguishing PSP-RS and PSP-P within the frontal pole, inferior frontal gyrus. The more significant loss was observed in PSP-RS. Additionally, more severe cortical atrophy was observed in the globus pallidus, amygdala and thalamus. The thalamocortical volume loss was interpreted as a feature of PSP-RS, however no correlation between this atrophy and the presence of typical PSP symptoms was found. The atrophy of supramarginal gyrus did not differ PSP-RS from PSP-P (5). Pathological studies based on 8 patients with PSP-P and 10 with PSP-RS, showed differences in the involvement of substantia nigra. In PSP-P neuronal loss with gliosis was found in 87.5% of patients, whereas in PSP-RS in 50% of patients. This group also examined the neuronal loss with gliosis within the subthalamic nucleus, which was also significantly more common among PSP-P patients (6). Another work based on 18 patients with PSP-RS and 12 patients with PSP-P showed different patterns of tau pathology (7). Changes observed in PSP-RS were more disseminated. Significant differences were observed within the basal ganglia, subthalamic nucleus, tectum, locus coeruleus and dentate nucleus. Another work indicating PSP-tau score (12 grade—*0 mild tau pathology*, >*7 widespread pathology*) revealed no cases of PSP-P with PSP-tau score above 5. The authors of this work found a negative correlation between the PSP-tau score and disease duration (37).

PSP-P analyses lack extended studies on a genetic basis. PSP is interpreted as a mainly sporadic disease, however certain works describe risk factors which may be associated with PSP such as H1 haplotype of *MAPT* and its subtype H1c (38). Growing interest concerning additional PSP risk alleles is related to the risk alleles in *STX6* and *EIF2AK3*. In a work describing a patient with PSP-P clinical phenotype, the authors showed the association with *VPS35* and *FBXO7* genes. Neuropathological examination revealed alfa synuclein Lewy body pathology. PSP-P clinical manifestation was associated with deterioration observed in the brain stem (39).

Analysis regarding differences in neurofilaments showed differentiation between PD and atypical parkinsonisms, which may be useful in distinguishing PSP-P and PD (40–42). No differences regarding differentiation of PSP phenotypes using this method were revealed.

The general conclusion of the additional examinations in PSP-P is that the obtained results do not provide specific differentiating tools. MRPI 2.0 and P/M ratio may be interpreted as an interesting feature possibly useful when analyzed as a correlation with clinical manifestation. The damage of WM supratentorial tracts in PSP-P seems to be an evolving tool, however contemporarly requiring more detailed analysis. Other MRI evaluations such as the assessment of mesencephalic tegmentum, superior cerebellar peduncle, dentate nucleus show more pronounced deterioration in PSP-RS. Volumetric analyses provide similar results when analyzing the frontal lobe, however various structures such as the corpus callosum do not show any differences which could be implanted in everyday clinical practice. The TCS in the critical differentiation of PSP-P and PD, which is the most difficult feature in contemporary examination, may not be interpreted as a useful tool, as the results of PSP-P and PD patients may be the same. The TMS is a method which requires extended analysis and further studies based on larger groups of patients. Undoubtedly the repeating result of the presented studies shows cerebellar inhibition as a vital component of PSP syndrome, however the differentiation of PSP-RS and PSP-P at this stage is indicated only in the transcallosal stimulation study. The overall observation of the presented pathological studies on the differences between PSP-P and PSP-RS show more conspicuous boundaries, especially when interpreting the cortical volume loss and tau dissemination. The main drawback is they cannot be done *in vivo*, which may become an obstacle in the future when individualized treatment will be initiated.

Among the works mentioned in this section of the manuscript, only works by Quattrone et al., Seki et al., Benussi et al., Dale et al., and Menšíková et al., were published after the release of contemporary criteria of diagnosis of PSP.

## Conclusion

- Neuroimaging and other types of additional examinations show, that even in advanced stages of PSP-P, significant overlaps with PD rather than PSP-RS can be observed (Table 1).
- The differences between PSP-RS and PSP-P in majority of the presented studies were not verified by neuropathological examination particularly due to the necessity of *in vivo* assessment.
- Possible evolution of PSP-P into PSP-RS remains a question of whether it is an actual evolution of one entity into another or if it is more likely related to primary misdiagnosis.
- Future directions in the examination of PSP should indicate early differential diagnosis of PSP-P and PD-PIGD. This may enable treatment of PSP-P in the stages, when the therapy would be the most beneficial. In our opinion this can be achieved by obtaining an algorithm of neuroimaging, which could multidimensionally present abnormalities in the structures with stressed changes e.g., cerebellar peduncles, corpus callosum, pons and midbrain. Morphological and Metabolic changes should be extended by the next generations of tau radiotracers which show improved specificity.

**Table 1:** Comparison of PSP-P, PSP-RS, and PD features.

**Method/feature**	**PSP-P**	**PSP-RS**	**PDPIGD**
MRI—Midbrain tegmentum atrophy	–	+	–^a^
MRPI 2.0	++	+++^b^	+
MRI—midbrain atrophy: • P/M ratio • MTEGM	No abnormalities, eventually: ↑ P/M ratio ↓ MTEGM	↑↑↑ P/M ratio ↓↓↓ MTEGM	No abnormalities
MRI-−3D T1: • pons/brain ratio • MRPI	↑pons/brain ratio ↑MRPI (precedes vertical supranuclear gaze palsy)	↑↑↑ pons/brain ratio ↑↑↑MRPI	Almost no abnormalities
MRI: SCP	Atrophy +	Atrophy +++	Normal
MRI: mesencephalic tegmentum, superior cerebellar peduncle, dentate nucleus SCP, thalamus decussation	Mean diffusivity↑, fractional anisotropy ↓, volume ↓ Altered microstructural integrity of the dentatorubrothalamic tract + ++	Mean diffusivity↑, fractional anisotropy ↓, volume ↓ Altered microstructural integrity of the dentatorubrothalamic tract +++ +++	No change
DT MRI	Damage of white matter supratentorial tracts	Damage of white matter supratentorial tracts^c^	Changes in olfactory regions and SN^d^
DT MRI deterioration of infratentorial white matter and thalamic radiations	+	+++	No data available
3 T MRI corpus callosum volumetry	Central part ↓ Midanterior part ↓	Central part ↓↓↓ Midanterior part ↓↓↓	Central part ↓ Midanterior part ↓
DaT SCAN	Striatal uptake↓↓ Putamen to putamen-to-caudate nucleus ratio ↑↑↑	Striatal uptake ↓↓↓ Putamen to putamen-to-caudate nucleus ratio ↑↑↑	Striatal uptake↓ Putamen to putamen-to-caudate nucleus ratio↑
Iodobenzamide scan	Striatal uptake↑	Striatal uptake↓	Striatal uptake↓
TCS: Hyperechogenic SN Hyperechogenic lenticular nucleus III ventricle	73% 67%	14% 67% Wider	90%^e^ Not observed^f^
TCS SN III ventricle	86% hyperechogenic	96% normal echogenicity, wider	90%^e^
TMS - transcallosal inhibition	+	+++	+
Volumetric analysis - Cortical volume loss	Frontal pole, inferior frontal gyrus +, Supramarginal gyrus+, Internal globus pallidus, amygdala, thalamus+	Frontal pole, inferior frontal gyrus + + +, Supramarginal gyrus+, Internal globus pallidus, amygdala, thalamus +++	Pattern specific for clinical phenotype^g^: - Bilateral orbitofrontal, anterior cingulate, and lateral and medial anterior temporal gyri - Bilateral occipital gyrus, cuneus, superior parietal gyrus, and left postcentral gyrus (more pronounced cognitive impairment)
Tau levels in CSF	+	+	α-synucleinopathy
Neuropathology- Neuronal loss and gliosis of SN	87.5%	50%	Vast majority^h^
Tau pathology	Limited	disseminated	α-synucleinopathy
Neurofilaments in CSF	↑	↑	Normal
Genetics	^*^MAPT H1 haplotype, *H1c subtype*, *STX6* *EIF2AK3* *VPS35* *FBXO7*	^*^MAPT H1 haplotype, H1c subtype, *STX6* *EIF2AK3*	
Other	***EXCLUDING FACTORS:** visual hallucinations, drug induced dyskinesias, autonomic dysfunction*		

* Risk factors.

*CSF, cerebrospinal fluid; DaT SCAN, dopamine transporter (DAT) single photon emission computerized tomography; DT MRI, diffusion tensor MRI; MRI, magnetic resonance imaging; MRI 3D T1, T1 weighted 3-dimensional magnetic resonance imaging; MRPI, magnetic resonance parkinsonism index 2.0; MTEGM, the length between the interpeduncular fossa and the center of the cerebral aqueduct at the midmammillary-body level; P/M ratio, pontine to midbrain tegmental areas ratio; PD, Parkinson's disease; PIGD, Postural Instability Gait Disorder; PSP-P, progressive supranuclear palsy—parkinsonism; PSP-RS, progressive supranuclear palsy—Richardson syndrome; SCP, superior cerebellar peduncle; SN, substantia nigra; TCS, transcranial sonography; TMS, Transcranial Magnetic Stimulation*.

a Kato et al. (43).

b Quattrone et al. (22).

c Agosta et al. (28).

d Atkinson-Clement et al. (44).

e Berg et al. (45).

f Smajlovic and Ibrahimagic (46).

g Uribe et al. (47).

h *Dickson (48)*.

## Author Contributions

PA and NM: study design, data collection, data interpretation, acceptance of final manuscript version, and literature search. DK and AF: data interpretation and acceptance of final manuscript version.

### Conflict of Interest

The authors declare that the research was conducted in the absence of any commercial or financial relationships that could be construed as a potential conflict of interest.
